# Feature extraction from customer reviews using enhanced rules

**DOI:** 10.7717/peerj-cs.1821

**Published:** 2024-01-31

**Authors:** Rajeswary Santhiran, Kasturi Dewi Varathan, Yin Kia Chiam

**Affiliations:** 1 Department of Information Systems, Faculty of Computer Science & Information Technology, Universiti Malaya, Kuala Lumpur, Malaysia; 2 Department of Software Engineering, Faculty of Computer Science & Information Technology, Universiti Malaya, Kuala Lumpur, Malaysia

**Keywords:** Opinion mining, Sentiment analysis, Aspect extraction, Product review, Customer review, Pattern-based rule

## Abstract

Opinion mining is gaining significant research interest, as it directly and indirectly provides a better avenue for understanding customers, their sentiments toward a service or product, and their purchasing decisions. However, extracting every opinion feature from unstructured customer review documents is challenging, especially since these reviews are often written in native languages and contain grammatical and spelling errors. Moreover, existing pattern rules frequently exclude features and opinion words that are not strictly nouns or adjectives. Thus, selecting suitable features when analyzing customer reviews is the key to uncovering their actual expectations. This study aims to enhance the performance of explicit feature extraction from product review documents. To achieve this, an approach that employs sequential pattern rules is proposed to identify and extract features with associated opinions. The improved pattern rules total 41, including 16 new rules introduced in this study and 25 existing pattern rules from previous research. An average calculated from the testing results of five datasets showed that the incorporation of this study’s 16 new rules significantly improved feature extraction precision by 6%, recall by 6% and F-measure value by 5% compared to the contemporary approach. The new set of rules has proven to be effective in extracting features that were previously overlooked, thus achieving its objective of addressing gaps in existing rules. Therefore, this study has successfully enhanced feature extraction results, yielding an average precision of 0.91, an average recall value of 0.88, and an average F-measure of 0.89.

## Introduction

The past decade has witnessed rapid growth in online businesses, particularly in e-commerce. According to recent statistics from the United States Census Bureau, there was a 32.4% increase in online sales in 2020 compared to 2019 (https://www.census.gov/). It is therefore increasingly difficult to refute the impact of online activities, such as marketing and e-reviews, on product sales. The Local Consumer Review Survey 2023, conducted among US-based customers, reveals that 98% of customers read online reviews, 37% leave positive reviews, and 6% leave negative reviews. This survey also indicates that 70% of customers trust online reviews, especially when they see positive feedback from strangers. Notably, a single negative review can deter up to 30 potential customers. Therefore, the emerging significance of online reviews in influencing customer purchase intentions is undeniable.

According to Mumuni et al. (2020), the relevance of product reviews significantly influences the worth of such reviews to customers. Customers perceive a review as relevant when it discusses the characteristics (also known as features) of a product or service they are interested in. This is because customers’ evaluation of a product is critically dependent on its features, such as price, durability, and customer service. Accordingly, through reviews, customers typically attempt to find out as much as possible about a product’s features, including the specific features being discussed, the benefits of these features, the advantages or disadvantages of a particular feature in the product, and others’ experiences with the feature. This information not only influences customers’ purchasing decisions but is also invaluable to businesses seeking feedback to enhance their products and services. Moreover, apart from widely emphasized product features, there may be non-product-domain features that customers consider crucial in their product selection and purchasing decisions. Ultimately, products developed with features following the ‘voice of the customer’ (VoC) have a higher chance of success (Cooper, 2019).

Therefore, businesses need to detect features and customer opinions regarding feature performance for further assessment and improvement of their marketing and sales strategies. In this regard, customer review documents serve as vital mediums for the analysis of customers’ experiences and expectations regarding the product features, they deem important. However, companies are overwhelmed by a massive volume of product reviews (Rana & Cheah, 2019), while customers find it troublesome to sift through all the reviews and identify relevant ones before making purchase decisions. To address this challenge faced by both companies and customers, numerous studies have examined product review analysis and proposed various approaches to detect features and associated opinions in reviews. Notably, opinion analysis has emerged to play a pivotal role in automating the processing of a vast array of review documents, providing essential insights to both businesses and customers.

In recent years, several studies have utilized rule patterns and dependency parsers to detect features, achieving promising results. However, it has been observed that when mining unstructured review documents, several drawbacks can affect feature detection performance, including spelling or grammatical errors and irrelevant noises. An alternative to dependency parsers, which have grammatical constraints, is a pattern-based approach (Pak & Günal, 2022). The pattern-based approach is suitable for processing unstructured review documents as it mirrors informal writing structures (Tubishat, Idris & Abushariah, 2021). However, it requires properly cleaned review documents to achieve optimal performance. Moreover, many pattern rules deployed in previous studies assumed that features are always represented by nouns and opinions by adjectives, potentially missing features and opinion words that do not strictly conform to these grammatical assumptions.

The focal aim of this study is to enhance the explicit extraction of features from customer review documents by extracting and analyzing only features paired with opinion words. By achieving this objective, this study makes two key contributions: (1) introducing a new set of pattern rules to extract features that have often been overlooked by existing rules, and (2) employing the best-evaluated part-of-speech (POS) tagging framework to increase the accuracy of parsed words. The structure of this article is as follows. ‘Related Works’ discusses related existing studies, while ‘Proposed Methodology’ provides details on the framework and methodology. ‘Results and Discussion’ presents the experimental work conducted on five publicly available datasets, and ‘Conclusions and Future Works’ concludes the article.

## Related Works

This section presents the latest research and current state of the literature on explicit feature extraction in opinion mining. In a recent study, Zhou, Tang & Zhang (2023) employed the Dempster-Shafer ranking algorithm to extract and identify important product feature-opinion pairs, thereby improving performance. They also conducted feature ranking based on bipartite weighted network calculations. On the other hand, Rana et al. (2021) utilized an opinion lexicon developed for the Urdu language to initially extract opinions and then extract features, ranking the related features based on the distance between features and opinions. This approach led to the identification of opinion-feature pairs. Due to limited resources for the Urdu language, data preprocessing deviated slightly from standard preprocessing steps. CLE (https://www.cle.org.pk/), a part-of-speech (POS) tag for the Urdu language, was used for POS tagging, and two different sets of opinion lexicons for Urdu were employed. The feature extraction in this study, based on rules, achieved a precision of 0.78 and a recall of 0.76.

Similarly, Pak & Günal (2022) identified features and their scores using sequential pattern-based rules extraction. The features with the highest scores were finalized, but unlike previous studies, the extracted features were pruned into a final list before opinions were extracted. In this study, the F-measure performance for opinion targets showed significant improvement. In another recent study, Tran, Duangsuwan & Wettayaprasit (2021) utilized two different types of datasets comprising product reviews for electronic and computer products. They proposed an aspect of knowledge-based generation using patterns (AKGPR) and further trimmed the extensive list of extracted features using keywords, Word2Vec, and a similarity threshold. AKGPR achieved higher performance for feature extraction from electronic datasets with a precision of 0.89 and F-score of 0.81.

Additionally, Khan et al. (2019) introduced an ensemble learning method that utilized multiple N-Gram combinations to extract features. They applied multiple filtration techniques such as IG, MRMR, CHI, GR, and GI, along with multiple classifiers including SVM, naive Bayes, and GLM. The final selection of features from multiple result sets was done using the majority voting technique. This study utilized datasets from Cornell movie reviews and product reviews from amazon.com. Notably, Khan et al. (2019) effectively resolved the issue of noisy features resulting from irrelevant feature extraction in baseline studies, significantly improving performance compared to prior methods.

Over the years, many research works have adhered to the common concept of extracting nouns as features and adjectives as opinions. Following the same assumption, Rana & Cheah (2019) employed a feature extraction approach where the researcher defines sequential patterns and derives rules from these patterns to extract explicit features. After preprocessing the datasets obtained from Hu & Liu (2004) and tagging the words using Stanford parser, Rana & Cheah (2019) applied the PrefixSpan algorithm with SPMF, a Java software for pattern mining (Fournier-Viger et al., 2014). Patterns generated by the algorithm were pruned, and the longest patterns related to the words in the sentences were selected. Rana & Cheah (2019) initially identified nouns in the sentences as features and then used sequential patterns to establish associations with opinion words. This approach yielded improved precision and F-measure scores compared to previous studies, paving the way for similar approaches to be used in future studies to extract implicit features.

Using Amazon datasets, Hu & Liu (2004) and Asghar et al. (2019) tested 10 new extended rules for feature extraction to enhance results, achieving a precision of 83.46 and an F-measure of 77.16. After feature extraction, similar features were grouped together before identifying polarity and performing opinion summarization. Semantic similarity for feature grouping was calculated using PMI scoring, whereas polarity for the feature-opinion pair was determined using SWN and intensifier word detection. In their study, Agerri & Rigau (2019) conducted sequence labeling in SemEval multilingual datasets from 2014, 2015, and 2016 to perform opinion target extraction. The sequence labeling was implemented using IXA pipes, and the Perceptron algorithm was employed to learn the supervised model. This language-independent model achieved the highest performance for English reviews, with a precision of 81.55, recall of 87.3%, and F-measure of 84.1%

On the other hand, Konjengbam et al. (2018) proposed a framework for extracting nouns using feature ontology. They employed POS tagging to identify nouns and, to prevent ontology overload, only considered features that frequently appeared above a set threshold. Features with low semantics were pruned based on semantic knowledge. Using datasets from Hu & Liu (2004), the researchers deployed two different algorithms: ontology using the semantic relationship (SemR) algorithm and ontology with the semantic similarity (SemS) algorithm. The SemR algorithm achieved a precision of 0.56 and a recall of 0.74 for automated feature extraction, while the SemS algorithm achieved 0.79 for both precision and recall for the same.

In 2017, Rana & Cheah (2016) proposed a two-fold method, using SPR, first for feature and opinion extraction and then for frequency-based pruning for the identification of frequently used features related to domain-dependent opinions. NGD scoring was used to retain irregular but important features. Experiments were conducted on Amazon datasets (Hu & Liu, 2004) and achieved a precision of 0.87 and a recall of 0.92. In addition, Samha & Li (2016) introduced dependency relations for feature and opinion extraction, including lemmatization at the beginning of the process to group similar features and opinions. During preprocessing, the Stanford Dependency Relations was employed to identify a syntactic parser for mapping relations. The study also utilized two opinion lexicons, Wilson, Weibe & Hoffman’s (2005) lexicon and the General Inquirer, to identify features and extract opinion words more accurately. This approach yielded better performance than baseline studies, with a precision of 0.83 and a recall of 0.87.

To address the issue of data sparsity, He et al. (2017) proposed a neural attention model using word co-occurrence patterns, known as ABAE, for feature extraction. They used a restaurant review dataset from CitySearch and achieved a precision of 85.7%, recall of 72.2%, and F1 scores of 77.5%. This approach was the first unsupervised neural approach to be introduced for feature extraction. Alternatively, Akhtar et al. (2017) prescribed an ensemble method based on PSO for feature extraction from restaurant and laptop reviews, attaining a precision of 87.1% and an F1 score of 84.5%. Khan & Jeong (2016), meanwhile, put forth a supervised lazy learning model utilizing syntactic rules extracted using RapidMiner. They generated linguistic patterns of reviews using POS tags with Pen Treebank annotation. Features and opinions were extracted using previously employed rules, and the extracted features were validated using a training corpus containing individual product feature lists. Opinion extraction was validated against the opinion lexicon, and the review’s orientation for the feature was determined using SentiWordNet. With these methods, Khan & Jeong (2016) were able to reach a precision of 0.81 and a recall of 0.82 using datasets from amazon.com (Hu & Liu, 2004).

Maharani, Widyantoro & Khodra (2015) presented a new set of rules, in addition to existing rules, for identifying features. They used product review datasets from Hu & Liu (2004) and employed the Stanford Parser for POS tagging of review sentences. Sentences were tokenized into bigrams and trigrams to facilitate pattern usage for feature extraction. The authors developed 11 new patterns as part of their research contribution.

The concept of hybrid patterns, referred to as Combined Pattern Based Noun Phrases (cBNP), was proposed by Khan, Baharudin & Khan (2014) based on the dependency between nouns and adjectives. The researchers proved that subjective adjectives are best suited for opinion words, with nouns linked to the adjectives being likely feature candidates. The study utilized a product review dataset from Hu & Liu (2004) and a re-annotated version of the same dataset by Ferreira, Jakob & Gurevych (2008). The former dataset was annotated using feature-opinion matches, while the latter dataset was labeled using features relevant to the product. Khan, Baharudin & Khan (2014) also employed an opinion lexicon (Hu & Liu, 2004) to validate the extracted opinions and their polarity. This study achieved a precision of 0.79 and a recall value of 0.72.

Noun-based extraction was further improved by constructing patterns for feature derivation. Many researchers have begun using patterns and rules-based approaches for feature and opinion extraction. Htay & Lynn (2013), for example, proposed a new set of rules for feature extraction derived from patterns containing opinion words. POS tagging was used to identify nouns, followed by the use of domain-specific features for mapping *via* a manually tagged training corpus. Datasets for the experiments were obtained from amazon.com and epinion.com, and analysis indicated a precision of 0.73 and a recall of 0.86. Apart from extracting features, Htay & Lynn (2013) aimed to provide opinion summarization as well.

Moreover, Ravi Kumar & Raghuveer (2013) proposed deriving typed dependencies and collapsed dependencies, first using the LingPipe Sentence Boundary method to identify relevant sentences and then, using the Stanford Parser to parse the sentences. Eventually, the output of the parser would be deployed to extract features. The researchers also used words from the opinion lexicon (https://mpqa.cs.pitt.edu/lexicons/) and General Inquirer (https://inquirer.sites.fas.harvard.edu/) to seed the list of negative and positive words to identify opinion words. Experimenting with review datasets from amazon.com and cnet.com, their results demonstrated a precision of 0.73 and recall of 0.82.

In a fully unsupervised method, Bagheri, Saraee & de Jong (2013) employed iterative bootstrapping with an initial seed list expanded into a larger set. They then used A-score metrics and feature pruning to finalize the list of features and opinion words. Review sentences were tokenized into bigrams and trigrams before employing heuristic patterns. Features not associated with any opinion words or stop words were explicitly removed. This method significantly improved precision and recall compared to baseline studies, achieving a precision of 0.86 and a recall of 0.64.

In order to describe the syntactic relationship, Qiu et al. (2011) applied dependency grammar, which identifies the relationship between two words as either direct dependency or indirect dependency. Preprocessing was done using the Stanford parser and MINIPAR (https://gate.ac.uk/releases/gate-7.0-build4195-ALL/doc/tao/splitch17.html). The study used an opinion lexicon (https://www.cs.uic.edu/ liub/FBS/sentiment-analysis.html#lexicon) for seed purposes to initiate bootstrapping and propagation of opinion and extracted features. Considering only nouns as features and adjectives as opinions, Qiu et al. (2011) expanded the lexicon using newly found opinion words until no more feature or opinion words could be identified. The proposed double propagation method outperformed baseline studies using the same datasets (Hu & Liu, 2004), achieving a precision of 0.88 and a recall of 0.83. However, the expanded lexicon was not made available to the public.

On the other hand, Popescu & Etzioni (2007) extracted syntactic dependencies using the MINIPAR (https://gate.ac.uk/releases/gate-7.0-build4195-ALL/doc/tao/splitch17.html) parser. They used an unsupervised method called OPINE, which retained relevant features by applying frequency filtration and further assessed noun phrases using PMI score calculation.

Although the aforementioned pattern approaches have introduced numerous rules, their performance in related studies thus far indicates room for improvement in feature extraction efficiency. Therefore, this study aims to enhance feature extraction efficiency by: (1) introducing additional rules to extract more accurate features, (2) improving extraction through comprehensive preprocessing to generate cleaner documents, and (3) employing limited but efficient rules for feature extraction to avoid extracting a larger feature set, which may lead to performance degradation. To measure the performance of the proposed approach in this study, several studies using pattern rules for feature extraction are used as a reference, as detailed in Table 1.

**Table 1:** Analysis of past studies of feature extraction using pattern rules.

Studies	Feature extraction methods	Datasets	Domain	Results
Pak & Günal (2022)	Sequential pattern-based rule mining	Hu & Liu (2004); SemEval 2014 (Task 4)	Electronic Products/Restaurant	F1: 70.0%
Rana et al. (2021)	Syntactic rules with opinion lexicon for Urdu language	Mukhtar et al. (2018)	Urdu opinion texts	P:78.0%, R: 76.0%, F1: 76.0%
Tran, Duangsuwan & Wettayaprasit (2021)	Aspect knowledge-based generation using pattern rules (AKGPR)	Hu & Liu (2004); Liu et al. (2016)	Electronic/ Computer products	P:89.0%, R: 76.0%, F1: 81.0% P: 85.0%, R: 64.0%, F1: 73.0%
Tubishat, Idris & Abushariah (2021)	Heuristic Patterns, Whale Optimization Algorithm and Pruning	Hu & Liu (2004); Liu et al. (2015)	Electronic/computer products	P: 92.0%, R: 93.0%, F1: 92.0%
Chauhan & Meena (2020)	Domain-Specific aspect term extraction	Hu & Liu (2004)	Electronic Products	P: 88.0%, R: 85.0%, F1: 86.0%
Rana & Cheah (2019)	Sequential Pattern Rules using PrefixSpan algorithm with SPMF	Hu & Liu (2004)	Electronic Products	P: 86.0%, R: 91.0%, F1: 89.0%
Kang & Zhou (2017)	Extended DP with additional new rules	Hu & Liu (2004)	Electronic Products	P: 87.0%, R: 88.0%, F1: 87.0%
Liu et al. (2016)	Extended DP with Simulating Annealing	Hu & Liu (2004); Liu et al. (2015)	Electronic/ computer products	P: 85.0%, R: 91.0%, F1: 88.0%
Qiu et al. (2011)	Double Propagation (DP) using dependency rules and pruning	Hu & Liu (2004)	Electronic Products	P: 88.0%, R: 83.0%, F1: 86.0%
Khan et al. (2019)	EnSWF: POS and ngram-based ensemble method	Pang, Lee & Vaithyanathan (2002); Blitzer, Dredze & Pereira (2007); McAuley & Leskovec (2013)	Movie, Book, DVD, Electronics and Kitchen	Accuracy 91.64%
Asghar et al. (2019)	Heuristic patterns and lexicons	Hu & Liu (2004)	Electronic Products	P: 83.0%, R: 71.0%, F1: 77.0%
Agerri & Rigau (2019)	OTE using sequence labelling	SemEval 2014 SemEval 2015 SemEval 2016	Customer Reviews	(SemEval 2014): P: 81.5%, R: 87.3%, F1: 84.1% (SemEval 2015): P: 72.9%, R: 69.0%, F1: 70.9% (SemEval 2016): P: 73.3%, R: 73.7%, F1: 73.5%
Konjengbam et al. (2018)	Aspect Ontology	Hu & Liu (2004)	Electronic Products	P: 79.0%, R: 79.0%, F1: 79.0%
Rana & Cheah (2017)	Two-fold-rule based method	Hu & Liu (2004)	Electronic Products	P: 87.0%, R: 92.0%, F1: 89.0%
Akhtar et al. (2017)	PSO based ensemble learning method	SemEval 2014	Customer Reviews	P: 87.1%, R: 82.1%, F1: 84.5%
He et al. (2017)	Word embedding models with attention mechanism	Citysearch corpus	Restaurant Reviews	P: 85.7%, R: 72.2%, F1: 77.5%
Samha & Li (2016)	Dependency relations and lexicon	Hu & Liu (2004)	Electronic Products	P: 83.0%, R: 87.0%, F1: 77.0%
Khan & Jeong (2016)	Lazy learning model using syntactic rules	Hu & Liu (2004)	Electronic Products	P: 81.0%, R: 82.0%
Maharani, Widyantoro & Khodra (2015)	Pattern based extraction with new set of rules for explicit features	Hu & Liu (2004); Ding, Liu & Yu (2008)	Electronic Products	P: 62.6.0%, R: 72.8.0%, F1: 67.2%
Khan, Baharudin & Khan (2014)	Combined Pattern Based Noun Phrases (cBNP)	Hu & Liu (2004); Ferreira, Jakob & Gurevych (2008)	Electronic Products	P: 79.0%, R: 72.0%: F1:75.2%
Htay & Lynn (2013)	Pattern based extraction with new set of rules	Hu & Liu (2004)	Electronic Products	P: 73.0%, R: 86.0%, F1:79.0%
Ravi Kumar & Raghuveer (2013)	Dependencies using LingPipe Sentence Boundary, Lexicon and GI	amazon.com cnet.com	Customer Reviews	P: 73.0%, R: 82.0%
Bagheri, Saraee & de Jong (2013)	Iterative bootstrapping using rules and pruning	Hu & Liu (2004)	Electronic Products	P: 86.0%, R: 64.0%, F1:73.0%

## Proposed Methodology

**Figure 1:**
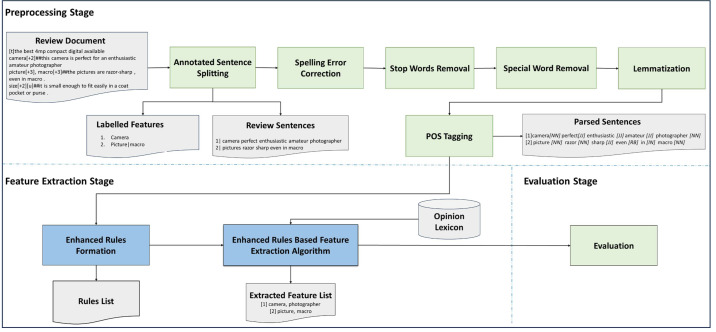
Methodology for feature extraction.

This study’s proposed methodology for feature extraction from review datasets is depicted in Fig. 1. The feature extraction steps comprise preprocessing and feature extraction phases using rules. The details of each phase are discussed in the following sub-sections.

### Tools and resources

The experiments conducted in this study were developed and executed using Python, which involved the usage of common Python libraries such as NLTK, NumPy, panda, and CSV. Additionally, Jupiter Notebook was utilized as a developer tool on Windows 10 Pro, whereas model and data libraries from the Flair (https://github.com/flairNLP/flair) framework (Akbik et al., 2019) were employed for POS tagging. The experiment codes (https://github.com/rajeswary3/feature-extraction) (https://doi.org/10.5281/zenodo.8365767) developed for this study are publicly available for reusability purposes.

### Dataset

Electronic product review datasets were used in this experimental setup, consisting of five products: two digital cameras, a cell phone, an mp3 player, and a DVD player. These customer review datasets (https://www.cs.uic.edu/ liub/FBS/sentiment-analysis.html#datasets) were developed by Hu & Liu (2004) and are publicly available. Further descriptive details of these datasets are provided in Table 2. Mainly, these datasets were chosen as they fit the research scope for the following reasons:

• They are open datasets.
• They were extracted from review domains and are not based on microblogs or Twitter.
• They have been used in many recent related studies (refer to Table 1).
• Opinionated product features are more tangible in nature, suiting explicit extraction.

In order to comprehend the actual performance of the proposed approach, it is important to compare results from studies that use the same product review datasets and feature extraction technique. The main reason for limiting the research scope as such is to prove the performance is being fairly compared in the same controlled environment that executes on the same product domain datasets with same size and structure.

**Table 2:** Dataset breakdown by number of review statements.

**Dataset**	**Reference**	**No. of review statements with labelled features with opinion**
Canon digital camera	D1	238
Nikon digital camera	D2	159
Nokia cellphone	D3	265
Creative MP3 player	D4	720
Apex DVD player	D5	344

The review documents are in a structured format, with features, polarity scores, and types (implicit/explicit) for each review sentence labelled. The annotation for each sentence starts either with [t] to mark the review title or labeled features followed by “##” for review sentences. The features were labeled with positive or negative scores within the range of +3 for the strongest opinion and −3 for the weakest opinion. Implicit features, where the features were not present in the review sentences, were tagged [u] and [p]. Additional tags that may be useful for future studies, such as [s] for author suggestions and [cs] for product comparisons, were also part of the review documents. As this study focuses on explicit feature extraction, only relevant review sentences were extracted through preprocessing. Therefore, of the 3,945 review sentences examined, 1,726 reviews with labeled features were selected and processed.

A combination of existing rules and new rules was tested with an opinion lexicon and two different POS taggers. The scope of this research is to identify explicit features in opinionated content; thus, it is compulsory for a feature to be accompanied by an opinion, irrespective of the polarization of the opinion words. For results evaluation, both labeled and predicted features were used.

### Preprocessing stage

Preprocessing constitutes the first stage of this study’s proposed feature extraction methodology. As shown in Fig. 1, the preprocessing stage begins with the review documents being processed to extract only relevant review sentences that contain explicit features. For the preprocessing activity, only review contents were used, hence review titles and implicit features related sentences were removed as the scope of the research is to identify explicit features only. The objective of this omission is to improve the performance of explicit feature extraction.

**Table:** 

Example of review statement:	Outcome after Preprocessing
[1] [t]glad to own.	to be removed
[2]##i have had this phone for about 5 months.	to be removed
[3]battery[+2]##i treat the battery well and it has lasted.	to be used for further processing

Line [1] is tagged as title [t] of the review. Lines [2] and[3] are the contents of the review. However, in Line [2] , there are no features annotated by the author. Line [3] has “battery” as the feature. Hence Line [1] and Line [2] are removed during preprocessing.

Review sentences composed by customers in plain language typically reflect their preferences, opinions, prior expectations, and emotions regarding the purchase and usage of a product or service. These sentences may also contain noise, spelling errors, grammar mistakes, abbreviations, unwanted expressions, or extraneous information (Haddi, Liu & Shi, 2013). Thus, text cleaning, as part of preprocessing, is essential in ensuring a high-quality processing. Text cleaning involves removing spaces, special characters, and stop words, as well as stemming and lemmatization.

Accordingly, the review sentences underwent a cleaning process aimed at rectifying spelling errors. Correcting misspelled words is crucial, as inaccuracies in spelling can result in a loss of intended meaning and yield unreliable outcomes. Reference lists (http://introcs.cs.princeton.edu/java/44st/misspellings.txt) from a prior study (Krishnakumari & Sivasankar, 2018) were used as samples of misspelt words for correction. Another step in text cleaning is to remove stop words and special words. Stop words were removed based on the NLTK stop word (https://www.nltk.org/nltk_data/) library for the English language. Punctuations and special characters, such as “@” or “&”, were also eliminated as part of the cleaning exercise. Following this, lemmatization was performed on words in the sentences to identify their root terms.

As the final step in preprocessing, each review sentence in the document was tagged with POS labels. Specifically, singular and plural nouns were identified as NN/NNS, adjectives as JJ, various types of adverbs as AVB/RB/RBR/RBS, different types of verbs as VB/VBZ/VBD, prepositions as IN, determiners as DT, opinion words as O, and features as A. Various POS libraries are used in feature extraction-related studies to determine the impact of tagging on the accuracy of opinionated feature extraction. The NLTK (https://www.nltk.org/) POS Tagger and Stanford (https://nlp.stanford.edu/software/tagger.html POS Tagger are popular POS tagger libraries, while the Flair (https://github.com/flairNLP) framework is considered a simple-to-use tagger. This study initially used both the NLTK and Flair tagger libraries in Python. Based on the results discussed in ‘Results and Discussion’, the Flair framework was selected for the final analysis.

### Feature extraction stage

This section provides a comprehensive discussion of the processes and pattern rules utilized in this study. The second stage of processing involved feature extraction using a combination of rules employed in prior studies and newly devised rules, aimed at enhancing opinionated feature extraction results.

One of the initial opinion lexicons was introduced by Hu & Liu (2004). It has evolved and been continually updated since 2004, now comprising approximately 6,800 positive and negative words. To complement this lexicon, this study also utilized another lexicon authored by Almatarneh & Gamallo (2018). This supplementary lexicon (https://github.com/almatarneh/LEXICONS) contains extreme opinions that represent the most favorable or unfavorable assessments. Consequently, extreme words not found in Hu & Liu’s (2004) lexicon were adopted from the lexicon by Almatarneh & Gamallo (2018). These lexicons serve as valuable resources for validating the extracted opinion words.

Selected existing rules from prior studies have been identified and presented in Table 3. To achieve this study’s objective of improving feature extraction efficiency, additional rules were formulated to extract more precise features. Previous studies have predominantly focused on noun-based words as features, despite evidence that certain features are non-noun words, such as verbs. This means features can indeed be extracted from non-noun words, and opinions need not exclusively consist of adjectives. As such, new rules were designed in this study to address the issue of some features being overlooked due to not falling within the common noun category. While nouns are commonly associated with features and adjectives with opinions, the new rule set acknowledges the presence of outliers. Table 4 provides details on the new rules that were formulated based on an analysis of sample datasets and observation techniques, while Algorithm 1 outlines the steps used for extracting features. Further experiments were conducted on robust datasets to validate the newly developed rules. All the codes developed for the experiment are publicly available on the authors’ GitHub (https://github.com/rajeswary3/feature-extraction) (https://doi.org/10.5281/zenodo.8365767).

**Table:** 

**Algorithm 1:** Feature extraction algorithm based on rules and opinion lexicon
**Input:** POS-tagged pre-processed sentence, feature-rule-lexicon, Opinion Lexicon
**Output:** Feature
Begin
1 for each POS-tagged-sentence “si” in POS-tagged sentences do
begin
2 while not (feature-rule-lexicon.eof)
begin
3 if (POS-tagged-sentence “si” matches rule in feature-rule-lexicon) then
begin
4 if (sentiment matches Opinion Lexicon) then
begin
5 Extract feature-sentiment N-gram along with rule # from feature-rule-lexicon
6 Store it in Feature-Opinion-Lexicon (L1)
End if
End If
End while
End for
End

### Evaluation stage

In this study, the evaluation criteria were set in reference to Liu et al. (2016), whereby precision, recall, and F-measure were calculated using TP (true positive), FP (false positive), and FN (false negative) values. Precision denotes the percentage of correctly identified features out of the total identified features, whereas recall identifies the percentage of identified features out of the total labelled features. The analysis of extraction is based on Table 5.

**Table 3:** Feature extraction pattern rule from existing studies.

**No**	**First word**	**Second word**	**Third word**	**Study**
1	AVB	JJ	NN/NNS	Asghar et al. (2019)
2	NN	NN	NN/NN	Maharani, Widyantoro & Khodra (2015)
3	JJ	NN/NNS		Asghar et al. (2019); Maharani, Widyantoro & Khodra (2015)
4	JJ	NN	NN	Asghar et al. (2019)
5	JJ	JJ	NOT NN/NNS	Maharani, Widyantoro & Khodra (2015)
6	JJ	TO	VB	Asghar et al. (2019)
7	JJ	VB/VBN/VBD	NN/NNS	Maharani, Widyantoro & Khodra (2015)
8	NN	TO	NN/NNS –NN/NNS	Maharani, Widyantoro & Khodra (2015)
9	NN	[RB]	NN /VB[O]	Maharani, Widyantoro & Khodra (2015)
10	NN	IN	NN	Asghar et al. (2019); Maharani, Widyantoro & Khodra (2015)
11	NN	JJ		Asghar et al. (2019)
12	NN	NN/NNS	JJ	Asghar et al. (2019); Tubishat, Idris & Abushariah (2021)
13	NN	[IN+DT]+NN+[VBP]	JJ [O]	Tubishat, Idris & Abushariah (2021)
14	NN	VBZ-RB	JJ [O]	Tubishat, Idris & Abushariah (2021)
15	NN	VBZ	JJ [O]	Tubishat, Idris & Abushariah (2021)
16	NN	VBZ+RB+JJ[O]	NN	Asghar et al. (2019)
17	NN/NNS	IN	DT –NN/NNS	Asghar et al. (2019)
18	NN/NNS	JJ	NOT NN/NNS	Tubishat, Idris & Abushariah (2021)
19	NN/NNS	JJ		Asghar et al. (2019)
20	NN/NNS/RB/RBR/RBS	JJ/VBN/VBD		Asghar et al. (2019)
21	PRP	VB	DT+NN	Asghar et al. (2019)
22	RB/RBR/RBS	JJ	NN/NNS	Htay & Lynn (2013)
23	VB	NN/NNS		Asghar et al. (2019)
24	VB	JJ		Asghar et al. (2019)
25	VB	VB	NN	Asghar et al. (2019)

**Notes.**

NN/NNS Singular/Plural Nouns JJ Adjectives AVB/RB/RBR/RBS Different types of Adverbs VB/VBZ/VBD Different types of Verbs IN Prepositions DT Determiner O Opinions A Features

**Table 4:** Newly formed feature extraction pattern rules.

**ID**	**First word**	**Second word**	**Third word**	**Example**
R1	JJ(A)	VB(O)		***infrared*** *blessing*
R2	NN	NN(O)		***vibration*** *top*
R3	RB(O)	NN		*better* ***speakerphone*** *ever*
R4	JJ(O)	JJ		*incredibly crappy* ***remote ;*** *pretty* ***sturdy***
R5	VB	JJ(O)		***screen*** *great ;* ***read*** *seconds*
R6	NN	RB(O)		*treat* ***battery*** *well last*
R7	JJ(O)	VB/JJ	NN	*outstanding signal* ***reception***
R8	JJ	RB	NN/NNS	*shooting scene tough automatically* ***focus***
R9	NN	RB +VBP	NN[O]	**case** ever make quality
R10	NN	RB[O]	NN	**controls** especially scroll wheel
R11	NN	RB	NN/JJ[O]	**backlit screen** infinitely better
R12	NN/NNS	VB/IN/NN/NNS	NN/NNS[O]	**software** getting favorite
R13	NN/NNS	VB/IN/NN/NNS	NN/NNS/JJ [O]	**scroll wheel** select push straight
R14	VB [A]	IN-DT/IN	JJ [O]	**read** within seconds
R15	JJ[O]	NN -VB	NN/NNS	best bet looking ** phone**
R16	NN	VB-RB	JJ[O]	**speaker** makes even great

**Notes.**

NN/NNS Singular/Plural Nouns JJ Adjectives AVB/RB/RBR/RBS Different types of Adverbs, VB/VBZ/VBD Different types of Verbs IN Prepositions DT Determiner O Opinions A Features

**Table 5:** Confusion matrix to evaluate feature extraction performance.

	**Relevant**	**Irrelevant**
Detected features	True Positive (tp)	False Positive (fp)
Undetected features	False Negative (fn)	True Negative (tn)

Precision, recall, and F-measure calculations (Liu et al., 2016) were formulated as Eqs. (1), (2) and (3) below:

(1) \begin{eqnarray*}\text{Precision},\mathrm{p}& = \frac{\text{Extracted features}\cap \text{Labelled features}}{\text{Extracted features}} \end{eqnarray*}

(2) \begin{eqnarray*}\text{Recall},\mathrm{r}& = \frac{\text{Extracted features}\cap \text{Labelled features}}{\text{Labelled features}} \end{eqnarray*}

(3) \begin{eqnarray*}\mathrm{F}-\text{Measure}& = \frac{2\mathrm{pr}}{\mathrm{p}+\mathrm{r}} .\end{eqnarray*}

The experiments were conducted on five different datasets, and performance was individually assessed before calculating the average results for the overall product datasets. Dataset details are provided in Table 2.

## Results and Discussion

Two sets of experiments were conducted. The first experiment focused on selecting the most suitable POS tagging framework, while the second experiment involved implementing the feature extraction rules proposed in this study. The following sections provide a detailed discussion of the experimental results obtained from both experiments. A complete end to end process illustration that describes sample input, output and evaluation is shown in Fig. 1.

### POS tagging framework selection

To determine the most suitable POS tagging framework for this proposed method, experiments were conducted on each of the individual products to evaluate the performance of two different tagging approaches: NLTK tagging and Flair tagging. The graphs below illustrate the results of precision, recall, and F-measure, demonstrating that the Flair tagging framework outperformed NLTK tagging. The Flair framework is a modern open-source NLP library built on PyTorch. It consistently exhibited superior performance to the NLTK library when applied to various product review documents. Therefore, subsequent feature extraction experiments utilizing rules were executed using the Flair framework.

### Feature extraction

Table 6 presents the overall performance of the proposed feature extraction method. Feature extraction experiments involved two sets of rules. The first set of rules consisted of existing rules used in prior studies, as presented in Table 3. The second set of rules combined the existing rule sets (Table 3) with a new set of rules (Table 4) formulated in this study. Table 7 showcases the results obtained using both sets of rules. It is evident that average precision improved by 6%, average recall by 6%, and the average F-measure value by 5% with the inclusion of the new rules introduced in this study. These new rules led to the identification of more features, directly enhancing the precision and recall of the study. The superior performance results in terms of precision and recall are attributed to the high data quality, which is a result of extensive data cleansing during the preprocessing stage.

**Table 6:** Performance obtained for feature extraction.

**Product**	**Performance**		
	**Precision**	**Recall**	**F-Measure**
D1	0.94	0.91	0.92
D2	0.92	0.92	0.92
D3	0.94	0.91	0.92
D4	0.91	0.88	0.90
D5	0.82	0.80	0.81
Average	**0.91**	**0.88**	**0.89**

**Notes.**

Bolded values indicate the average results of five datasets for this study.

**Table 7:** Comparing feature extraction performance by using different sets of rules.

Datasets	Rules from previous studies			This study		
	**Precision**	**Recall**	**F-Measure**	**Precision**	**Recall**	**F-Measure**
D1	0.88	0.85	0.87	0.94	0.91	0.92
D2	0.82	0.80	0.81	0.92	0.92	0.92
D3	0.90	0.87	0.89	0.94	0.91	0.92
D4	0.84	0.81	0.83	0.91	0.88	0.90
D5	0.80	0.77	0.79	0.82	0.80	0.81
Average	0.85	0.82	0.84	**0.91**	**0.88**	**0.89**

**Notes.**

Bolded values indicate the average values obtained in this study.

Further comparisons were made with previous studies that used rules for feature extraction on the same datasets as this study. Performance comparisons were made based on precision, recall, and F1-measure, as presented in Table 8. Based on the results, it can be observed that this study has set a new standard for pattern-based studies in terms of the number of rules and performance achieved. This study notably achieved the highest precision and F-measure for explicit feature extraction among all past studies.

**Table 8:** Results comparison with past studies in feature extraction using customer review datasets.

**Studies**	**Precision**	**Recall**	**F-Measure**	**Remarks**
**This study**	**0.91**	**0.88**	**0.89**	**41 pattern-rules**
Pak & Günal (2022)	NA	NA	0.70	NA
Tran, Duangsuwan & Wettayaprasit (2021)	0.89	0.76	0.81	20 pattern-rules
Tubishat, Idris & Abushariah (2021)	0.75	0.97	0.84	126 rules without the optimizer
Tubishat, Idris & Abushariah (2021)	0.92	0.93	0.92	57 rules with optimizer
Chauhan & Meena (2020)	0.88	0.85	0.86	Noun based extractions
Rana & Cheah (2019)	0.86	0.91	0.89	10 pattern-rules
Asghar et al. (2019)	0.83	0.71	0.77	10 pattern- rules
Rana & Cheah (2017)	0.87	0.92	0.89	10 pattern-rules
Kang & Zhou (2017)	0.87	0.88	0.87	7 dependency rules with 8 patterns
Samha & Li (2016)	0.83	0.87	0.77	16 dependency rules
Liu et al. (2016)	0.85	0.91	0.88	8 pattern-rules
Khan & Jeong (2016)	0.81	0.82	0.8	9 pattern-rules
Maharani, Widyantoro & Khodra (2015)	0.63	0.73	0.67	24 pattern-rules
Khan, Baharudin & Khan (2014)	0.79	0.72	0.75	16 pattern-rules
Htay & Lynn (2013)	0.73	0.86	0.79	8 pattern-rules
Bagheri, Saraee & de Jong (2013)	0.86	0.64	0.73	4 pattern-rules
Qiu et al. (2011)	0.88	0.83	0.86	4 pattern-rules

**Notes.**

Bolded values indicate the results obtained for this study.

Table 9 provides a detailed comparison of the performance for each dataset used in this study against the study that attained the highest performance in the past. While Tubishat, Idris & Abushariah (2021) achieved the highest values since publication in 2021, the subsequent study by Tran, Duangsuwan & Wettayaprasit (2021) reported lower results. An important aspect to highlight here is that Tubishat, Idris & Abushariah (2021) employed additional tasks and resources, such as the product manual and optimization techniques, to enhance feature extraction performance. Although they mentioned conducting pruning based on the product manual, there is limited clarity on how the product manual or user guide was used in the research to detect product features. Detecting feature frequency with a certain threshold (*e.g.*, a threshold of two for single-word features) within the product manual may appear ambiguous when considering the entire guide is to be interpreted and processed word by word. Attempts to contact the authors for clarification were unsuccessful.

**Table 9:** Feature extraction performance - precision, recall and F-Measure comparison between baseline studies and this study by dataset.

**Dataset**	**Tubishat, Idris & Abushariah (2021)**			**This study**		
	**Precision**	**Recall**	**F-Measure**	**Precision**	**Recall**	**F-Measure**
D1	0.91	0.93	0.92	**0.94**	**0.91**	**0.92**
D2	0.91	0.92	0.91	**0.92**	**0.91**	**0.92**
D3	0.93	0.93	0.93	**0.94**	**0.91**	**0.92**
D4	0.93	0.95	0.94	**0.91**	**0.88**	**0.90**
D5	0.92	0.91	0.91	**0.82**	**0.80**	**0.81**
Avg	0.92	0.93	0.92	**0.91**	**0.88**	**0.89**

**Notes.**

Bolded values indicate the results obtained for this study.

Further to that, despite the successful implementation of optimization techniques in Tubishat, Idris & Abushariah’s (2021) research, this study did not consider optimization techniques for several reasons. According to Osaba et al. (2021), optimization needs to be evaluated based on various criteria, including processing time, memory requirements, and the time needed to obtain results, particularly in an environment with significant computational demands. Tubishat, Idris & Abushariah (2021) aimed to optimize and select the best subset of rules out of 126 rules defined using a training dataset. As a result of their LSA algorithm, a set of 57 rules was frequently selected as the best. Hence, their decision to utilize an optimization algorithm was justified. In contrast, in this study, only a set of 41 rules (both existing and new rules) was used. The decision not to employ optimization in this study thus saved memory and processing time.

Lastly, when Tubishat, Idris & Abushariah (2021) applied rules without optimization techniques, they achieved a precision of 0.75 and an F-measure of 0.84. Comparatively, the experiment results from this study demonstrated higher precision than all baseline studies, indicating that the study successfully achieved its research objective. It is also established that features for extraction are not necessarily limited to nouns, and opinions are not solely adjectives. They can encompass other linguistic categories as long as they reflect a specific property or characteristic of a product or service.

An extensive review was made of all the recent studies involving pattern-based feature extraction. This is to identify the gaps that could be addressed by the proposed approach. The review of all the recent related studies (related to rule-based extraction) is crucial to ensure there is no repetitive effort in the same field/technique. These findings have provided a valid problem statement that is very much focused on rule-based feature extraction. There are a few reasons why no comparison is made to other types of alternative techniques.

(a) Firstly, the scope of research was focused on rule-based techniques to ensure that the approach can provide a solution in improving feature extraction performance by addressing a problem statement within the field/technique. Hence all the results evaluations were made against research that also used rule-based techniques.
(b) The research objective is to improve the feature extraction performance, where undoubtedly the improvisation is more justifiable when comparing results and performance produced by different studies within the same field. This study does not mean to identify which technique yields better performance.
(c) Alternate techniques, such as deep learning or improved optimization techniques may require larger data points and training datasets, increased setup cost and time, powerful hardware setup and complex algorithms. Comparing extraction performance produced from such a complex and significant computational setup is not fairly justified. Results produced by such deep learning methods are probably much better upon execution, however, the investment in setting up a proper environment for execution is comparatively huge. As a reference to Tubishat, Idris & Abushariah (2021), who have used optimization on top of rule-based mining techniques have better results upon optimization. However, this resulted in more memory and time consumption.
(d) In addition to (c), evaluating the performance produced with different techniques, which were executed under different experimental setups and parameters, may not be a fair comparison.

According to the Kano model, businesses must revolutionize their strategy in identifying customer needs, determining essential product features to meet those needs, and rectifying product deficiencies. This transformation necessitates a shift in how customer feedback is collected and analyzed, moving away from the traditional approach of waiting for customer complaints or using formal feedback channels. Rather, customer reviews serve as a crucial marketing tool, attracting more customers and aiding newcomers in making informed decisions about product performance. An effective evolutionary approach is to leverage computational assistance in mining customer reviews. The challenge, however, lies in identifying relevant product features within these reviews. This problem has prompted numerous researchers to seek ways to detect as many product features as possible in review documents. To this end, this study’s proposed approach represents a successful attempt to identify features that attract customers but have been undiscovered by existing rules.

Many customer review management tools, such as Trustpilot or feefo.com, are available in the current market, assisting both businesses and customers. Similar tools can be developed to assist customers using the enhanced rules proposed in this study. Identifying accurately reviewed features will further enhance search engine optimization (SEO) credibility and, ultimately, foster customer trust in making informed decisions. Simultaneously, regression analysis can be conducted on the extracted features, particularly in terms of the correlation and dependence among them, to provide more impactful insights to customers. For instance, one can explore the relationship between the price and size of a hotel room, offering customers a better understanding of whether a higher price is justified when the room size is larger. Notably, the proposed approach in this study ensures a higher number of features are extracted, which is crucial to comprehensively assess the dependency and relationships between features.

**Figure 2:**
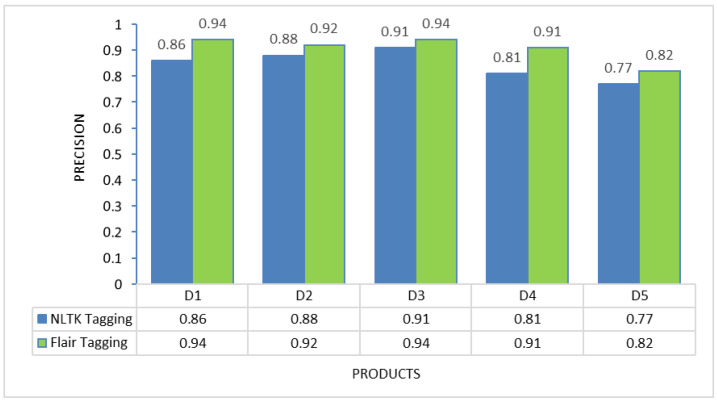
Precision comparison between NLTK tagging and Flair framework tagging.

**Figure 3:**
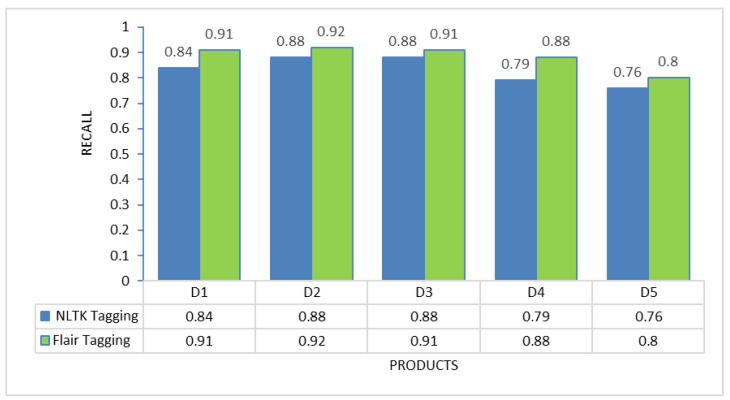
Recall comparison between NLTK tagging and flair framework tagging.

**Figure 4:**
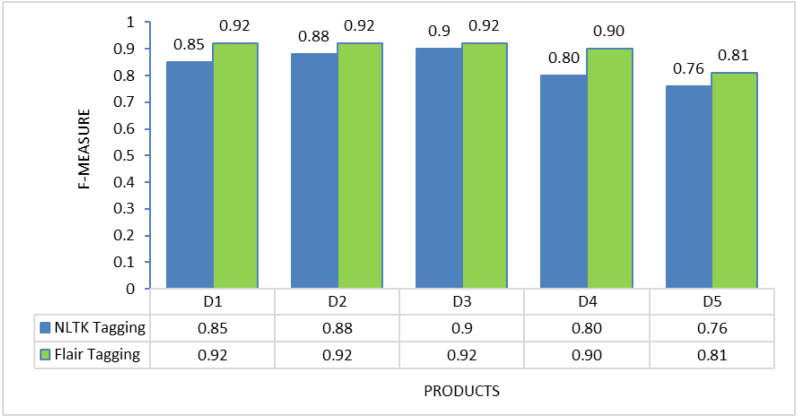
F-measure comparison between NLTK tagging and flair framework tagging.

Nevertheless, the challenge in implementing this proposed solution lies in the thorough preprocessing it requires. Review data cannot be used directly after extraction from review sites; instead, preprocessing activities, including noise removal and spelling error correction, are essential to present a clean review document for further processing. Additionally, as demonstrated in Figs. 2 to 4, the choice of different POS tagging frameworks may impact extraction performance. Hence, for the real-world implementation of this proposed approach, using the Flair framework for POS tagging is highly recommended.

## Conclusions and Future Works

The increasing focus on sentiment analysis and opinion mining highlights the significance of studying customer opinions for informed decision-making. The critical task in processing opinion documents retrieved from various sources is to accurately extract the message being conveyed. Often, automation may overlook subtle details, which calls for an enhanced extraction process. This shared objective has driven substantial research in the field of opinion mining. Among the numerous attempts, this study has successfully achieved its objective of improving feature extraction results by introducing additional rules designed to extract features that are missed by common heuristic patterns. The study has proposed a combination of 41 enhanced pattern rules, which together yield an average precision of 0.91, an average recall value of 0.88, and an average F-measure of 0.89 when applied to customer reviews. In the future, this research can be extended to explore improvements in implicit and comparative feature extractions. The proposed approach can also be tested on datasets from different domains to validate its robustness.

## Additional Information and Declarations

### Data Availability

The following information was supplied regarding data availability: The raw datasets used in this study are available from: Hu M and Liu B. 2004. Mining and summarizing customer reviews. In: Proceedings of the Tenth ACM SIGKDD International Conference on Knowledge Discovery and Data Mining. ACM, 168–177. https://doi.org/10.1145/1014052.1014073
The dataset: https://www.cs.uic.edu/ liub/FBS/sentiment-analysis.html#datasets. The experiment codes developed by the authors for this study are publicly available for reusability purposes at Zenodo: rajeswary3. (2023). rajeswary3/feature-extraction: Feature Extraction in Opinion Mining v1.0 (FeatureExtraction). Zenodo. https://doi.org/10.5281/zenodo.8365767.
